# Polio Outbreak Investigation and Response in The Horn of Africa: 2013-2016

**DOI:** 10.29245/2578-3009/2021/S2.1104

**Published:** 2021-04-07

**Authors:** Samuel Okiror, Abraham Mulugeta, Iheoma Onuekwusi, Fiona Braka, Sylvesta Malengemi, John Burton, Rustam Hydarav, Brigitte Toure, Bob Davis, Carolyn Gathenji, Chidiadi Nwogu, Joseph Okeibunor

**Affiliations:** 1 WHO Horn of Africa Coordination Office (HOA), Nairobi KENYA; 2 WHO, EMRO Amman; 3 WHO, Nairobi Kenya; 4 WHO Country Office, Nigeria; 5 WHO, Juba South Sudan; 6 UNHCR, Nairobi Kenya; 7 UNICEF Nairobi Kenya; 8 American Red Cross, Nairobi Kenya; 9 WHO Regional Office for Africa (WHO AFRO)

**Keywords:** Poliomyelitis eradication, Outbreak, Poliovirus, Horn of Africa

## Abstract

**Background:**

There has been civil strife, spanning more than two decades in some countries and recurrent natural disasters in the Horn of Africa (HoA). This has consistently maintained these countries in chronic humanitarian conditions. More important however is the fact that these crises have also denied populations of these countries access to access to lifesaving health services. Children in the difficult terrains and security compromised areas are not given the required immunization services to build their immunity against infectious diseases like the poliovirus. This was the situation in 2013 when the large outbreaks of poliovirus occurred in the HoA. This article reviews the epidemiology, risk, and programme response to what is now famed as the 2013-204 poliovirus outbreaks in the HoA and highlights the challenges that the programme faced in interrupting poliovirus transmission here.

**Methods:**

A case of acute flaccid paralysis (AFP) was defined as a child <15 years of age with sudden onset of fever and paralysis. Polio cases were defined as AFP cases with stool specimens positive for WPV.

**Results:**

Between 2013 and 2016, when transmission was interrupted 20,266 polio viruses were in the Horn of Africa region. In response to the outbreak, several supplementary immunization activities were conducted with oral polio vaccine (OPV) The trivalent OPV was used initially, followed subsequently by bivalent OPV, and targeting various age groups, including children aged <5 years, children aged <10 years, and individuals of any age. Other response activities were undertaken to supplement the immunization in controlling the outbreak. Some of these activities included the use of various communication strategies to create awareness, sensitize and mobilize the populations against poliovirus transmission.

**Conclusions:**

The outbreaks were attributed to the existence of clusters of unvaccinated children due to inaccessibility to them by the health system, caused by poor geographical terrain and conflicts. The key lesson therefore is that the existence of populations with low immunity to infections will necessary constitutes breeding grounds for disease outbreak and of course reservoirs to the vectors. Though brought under reasonable control, the outbreaks indicate that the threat of large polio outbreaks resulting from poliovirus importation will remain constant unless polio transmission is interrupted in the remaining polio-endemic countries of the world.

## Introduction

Despite the accelerated push on poliovirus made with the formation of the Global Polio Eradication Initiate (GPEI) to eradicate the poliovirus within a given timeframe, poliovirus outbreaks hit the Horn of Africa (HoA) between the periods of 2013 and 2014^1–6^. In 2013, an outbreak of wild poliovirus type 1 (WPV1) began in the Horn of Africa. Six cases were confirmed, four from Somalia (Banadir and Bay region) and two from Kenya (Dadaab in north-eastern Kenya). The first case was confirmed in Somalia on 9 May 2013 and in Kenya on 22 May. This was the first outbreak in Somalia since 2007 and in Kenya since 2011^7,8^. By 1 July 2013, 25 cases had been reported from Somalia (primarily from Banadir region) and six from Kenya (Dadaab in northeastern Kenya) and by 14 Aug 2013, Somalia had the worst outbreak reported globally in a non-endemic country with 105 cases confirmed. At the same period 10 cases of wild poliovirus were confirmed in Kenya while six cases of polio were confirmed in Ethiopia.

The series of outbreaks in Horn of Africa threatened the World Health Assembly (WHA)–endorsed Polio Eradication and Endgame Strategic Plan 2013–2018 objective of stopping all polio transmission globally by the end of 2014^9^. The GPEI team in the Horn of Africa responded to the outbreak of wild poliovirus type 1 (WPV1) with renewed determination to win the war against poliomyelitis in the Region, seemingly equal vehemence as the onslaught of the virus in the region^10^. The first vaccination campaign in response to the outbreak, reaching 440 000 children, took place 14-16 May 2013 in Somalia and a second round of vaccination was implemented as planned for 26to 29 May 2013 in synchronization with eastern parts of Kenya, targeting just over 1 million children.

Given the determination of the response team, the polio outbreak in the Horn of Africa started on the decline and the total number of polio cases stood at 203 (183 from Somalia, 14 from Kenya and six from Ethiopia) as at the end of 2013. However, by 18 Jun 2014, the total number of cases in the region was 223 since the beginning of the outbreak in Apr 2013 (199 from Somalia, 14 from Kenya and 10 from Ethiopia). Two new cases of circulating vaccine derived poliovirus type 2 (cVDPV2) were later confirmed in from Rubkona district of Unity State in South Sudan in the week of 4 September 2014.

All the same by the second half of 2014, the outbreaks in the Horn of Africa that spanned 2013 and the first half of 2014 were brought to the verge of being stopped and this was attributed to the regionally-coordinated outbreak responses. Immunization campaigns were planned and implemented to boost population immunity levels and minimize the risk of spread of the outbreak. The polio coordinating office and their teams in the Horn of Africa demonstrated unusual commitment to successfully implement polio eradication strategies. This paper documents and describes the 2013-2014 polio outbreaks in the 10 Horn of Africa countries with emphasis on the epidemiology, risk factors, response activities, and challenges and lessons learned in making the region polio free.

## Methods

### Setting and population

The Horn of African comprised of 10 countries, namely Djibouti, Ethiopia, Eritrea, Kenya, Somalia, and South Sudan. Others are Sudan, Tanzania, Uganda and Yemen. The Region has an estimated total population of 336,768,204 persons prior to the outbreak in 2013. Children aged <15 years make up 41.2% of the total population and those less than 5 years constituted 21.7% of the total population (Table 1). Political instability, insecurity, and recurrent natural disasters are major drivers of population movements within the Region; in 2013, an estimated 1.2 million internally displaced persons were reported within the Somali for instance^11^, the majority of whom often move into the capitals. The reported national coverage with 3 doses of oral polio vaccine (OPV3) through routine immunization has been historically low in some of the countries and was specifically <50% in 2012 in Somalia^12^.

### Acute Flaccid Paralysis (AFP) surveillance and case identification in HOA

Cases of polio are identified through the AFP surveillance system^7^. The polio surveillance network in the different countries, which make up the HoA, consist of national, regional, district levels, and health facility based surveillance officers with support from technical partners such as the World Health Organization (WHO) and the United Nations Children’s Fund (UNICEF). National staff members often have access to security-compromised areas of each country where international staffs have limited access.

**Figure 1:**
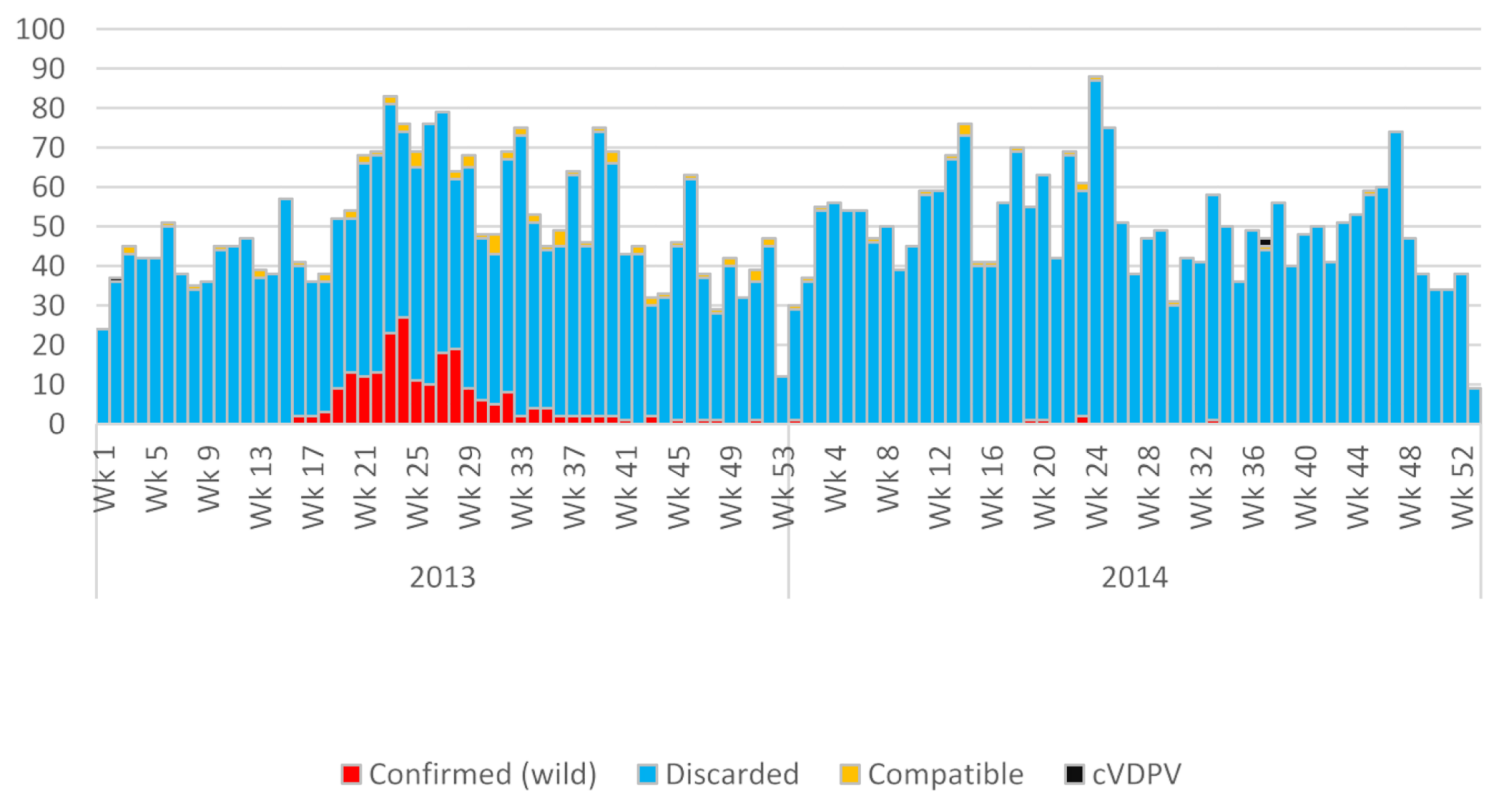
Reported AFP, wild poliovirus and cVDPV2 cases in Horn of Africa by Classification, 2013-2014

Surveillance officers visit on average 500 reporting sites, distributed across each country, on a weekly basis to actively search for cases of AFP (see Table 2). The surveillance network also extends to communities, through traditional healers, private pharmacies, vaccinators, and village polio volunteers, who feed into the national health systems. A case of AFP is defined as a child aged <15 years with sudden onset of fever and paralysis^7^. All AFP cases are investigated and stool samples collected by national polio workers according to AFP surveillance guidelines^13^. For each AFP case, 3 additional stool samples are collected from contacts. An AFP case contact is defined as anyone of similar age to the AFP case who resides in the same household or neighborhood. Stool samples collected from AFP cases and contacts in Eritrea, Yemen, Djibouti, Somalia and Kenya are sent to the Kenya Institute of Medical Research (KEMRI) polio laboratory for virus isolation, typing, and intratypic differentiation, using the WHO standard polio testing procedures^14^. South Sudan, Tanzania and Uganda are serviced by the laboratory in Uganda while samples from Ethiopia and Sudan were handled within the countries. WPV strains isolated by the polio laboratories were sent to the Centers for Disease Control and Prevention (Atlanta, GA) or the National Institute for Communicable Diseases (Johannesburg, South Africa) for genetic sequencing. Based on the outcome of the laboratory investigation, AFP cases are classified as either confirmed polio cases or discarded as cases of non–polio-associated AFP. Additional significant potential laboratory outcomes include classification of virus isolates as Sabin-like virus or vaccine-derived poliovirus (VDPV). Fig 1 summarizes AFP cases detected, wild polioviruses confirmed, circulating vaccine derived polioviruses confirmed.

### Data collection and analysis

Demographic, clinical, and laboratory information on AFP and confirmed polio cases are recorded in the AFP surveillance database. Analysis of AFP data is conducted by the programme on a regular basis to monitor AFP surveillance performance and the database is shared on a weekly basis with the Polio Eradication Initiative partnership for regional and global reporting. Additional sources of information for this report include supplementary immunization activity (SIA) data, outbreak response reports and action plans, monitoring reports and weekly bulletins.

As of 31 December 2013, stool samples from 545 AFP cases and 1761 contacts were collected and shipped to the KEMRI polio laboratory for WPV isolation. Since 2000, the countries have exceeded the WHO-established minimum AFP reporting rate of 2 non–polio-associated AFP cases per 100,000 children aged <15 years and has maintained key AFP surveillance indicators above certification standards at the national level^6^.

## Results

### Detection of the 2013 WPV outbreak

On 10th July 2013, 30th April 2013, 18th April 2013 and 9th September 2014 the first polioviruses of this outbreak were reported in Ethiopia, Kenya, Somalia and South Sudan respectively. While the first three were Wild Polio Virus type 1, it was a cVDPV2 in South Sudan. The Somalia case was a 32-month-old girl from Hamar Jabjab district in Mogadishu was confirmed as having WPV1 infection by the KEMRI polio referral laboratory in Nairobi (epidemiology week 15)^7^. The family had no known history of travel outside Mogadishu prior to onset of paralysis. In addition, 3 close contacts of the index case were confirmed to have asymptomatic WPV1 infection. A detailed case investigation was conducted immediately following the confirmation of the index case. See Figure 1.

**Figure 2:**
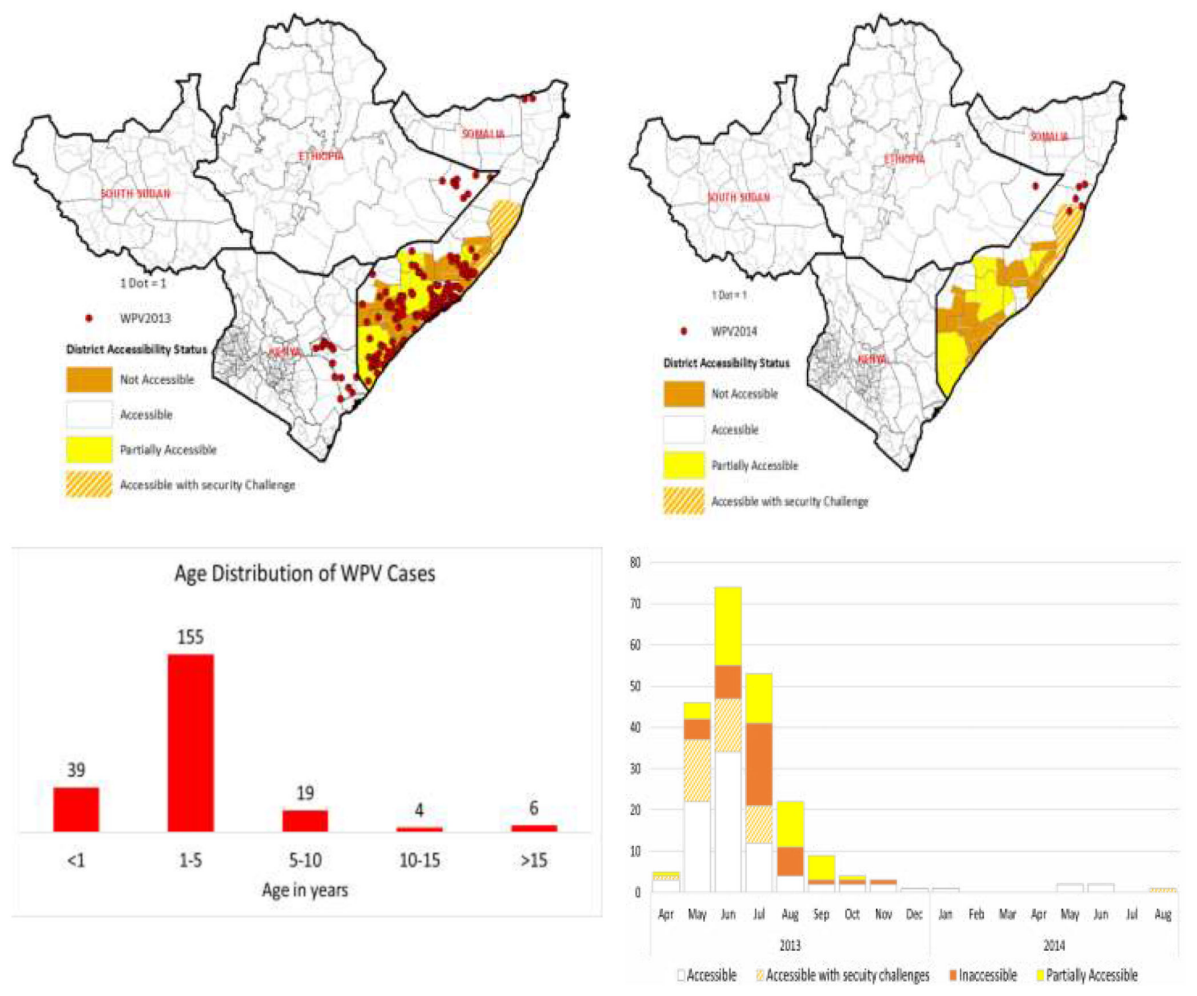
Geographical distribution of wild poliovirus (WPV) infections and district accessibility status in HOA during Outbreaks in 2013-2014.

For more than three years, millions of children aged <10 years could not be reached with OPV in some parts of HoA due to either geographical or security isolation. In Ethiopia and Kenya the reason for inaccessibility of the children were attributed to the difficulty of the terrain and very poor road network if any. On the other hand, the reasons for non-vaccination in Somalia and South Sudan were mainly due to inaccessibility linked to insecurity with the ongoing Al Shabab insurgency but also with very difficult terrain and poor road network. Some of the districts of South and Central Somalia, for instance could not be reached because of the ban imposed on immunization by some local antigovernment elements. Of the 109 districts listed in the AFP database, 27 are currently completely inaccessible for SIAs, while 12 are partially accessible^7^. Immunization campaigns could not be conducted in limited localities within the districts. Figure 2 shows the distribution of 223 wild poliovirus cases confirmed in 2013 and 2014, the age groups of the cases most (155) between 1 to 5 years (39) below one year of age, 19 between 6 and 10 years, 4 between 11 and 15 years and (96) above 15 years of age. Of the wild poliovirus cases confirmed, (43) were in inaccessible areas (54) in partially accessible areas, (39) in accessible areas with security challenges and (87) in accessible areas.

As a result of these outbreaks, surveillance activities were strengthened throughout the Horn of Africa. Active search for AFP cases through the extensive network of polio officers was intensified at reporting sites and within the communities, resulting in a marked improvement of the key AFP surveillance indicators at all levels. See Table 2.

### Outbreak response

Different approaches were implemented to respond to the outbreak. The known and most effective strategies to control the outbreaks and stop transmission were notably strong surveillance, large-scale polio supplementary immunization activities (SIAs) with OPV implemented as soon as possible after notification of the first case and continued on a large scale, and targeted SIAs using bivalent OPV until the outbreak was stopped. These activities were implemented at different times in the different countries.

### Immunization response

In Ethiopia there were a total of four national immunization days (NIDs), 18 sub-national immunization days (SNIDs), one Mop up defined as a round implemented in a limited area identified as poorly covered during the NIDs or sNIDs and 1 Emergency round defined as a round conducted in the immediate surrounding area of the confirmed case while preparations are still ongoing for a larger response. Furthermore there were a Permanent/ Transit Vaccination (PTV) points to cater for the mobile populations. A total of 44 PTVs were established in Somali Region, the outbreak region in Ethiopia and between 2014 and 2015 when they were operational, 69,401 children under 15 years were reached and less than 5% were “Zero Dose” ie never immunized before. Water point vaccinations were also conducted. In Kenya there were six NIDs, 14 SNIDs and 1 Mop up. In Kenya there was only one Permanent Vaccination point at the border with South Sudan, Somalia, there were 21 NIDs, 6 SNIDs, 5 Hard-to-Reach (HTR) vaccination points. Ten Short Interval Additional Dose (SIADs) and Child Health Days (CHD) were conducted. Permanent Transit Vaccination Points were established around the insecure areas to deliver vaccines to children who go in and out of the insecure areas as at August 2016 we had 406 permanent transit vaccination points mainly in South Central Zones which high insecurity. As at end of 2016, a total of 1,988,476 children under 10 years of age were vaccinated with 15,327 (0.77%) vaccinated for the first time. In South Sudan, 10 NIDs, 5 SNIDs and 4 SIADs were conducted. Next, there were permanent vaccination points were established around the insecure States of Upper Nile, Jungle and Unity to vaccinate children moving in and out of these areas, 26 such points were used to vaccinate 44,381 children under 15 years of age with OPV. Rapid Response Mission (RRM), which were being conducted for other services were also used for vaccination. Overall 54 such mission were conducted to various parts of the country between October 2014 and May 2016 reaching 257,469 children under 5 years with all antigens including OPV. This included 5 rapid response missions (RRM) to the County of Khorflus which was mostly inaccessible and 12,326 Children under 15 years of age were reached with all vaccinations including OPV.

## Coordination

Polio control and coordination rooms were established in Nairobi and in the outbreak countries to improve the efficiency of outbreak response operations. The control rooms improved the overall coordination of the outbreak response operation across partner organizations by maximizing communication, reducing bottlenecks, and streamlining reporting activities. Situation reports on the outbreak were shared with partners on a weekly basis through polio control rooms.

In other words, the overall coordination monitoring and evaluation was the responsibility of the HOA Coordination office and at that level the HOA office organized the Technical Advisory Group (TAG) meetings, which were held every 6 months, the TAG Teleconferences with all the HOA countries held in between the TAG meetings, outbreak response assessments (OBRAs), surveillance reviews, produce initially weekly updates then later when outbreak was over produced monthly, situation reports (SITREPs) produced monthly as well. The HoA office also held Country specific Coordination meetings the day before the TAG meeting to review country presentations, agree on activities to be synchronized, discuss operational challenges and management issues.

In Ethiopia, the coordination of the response was under the Minister of State who was the Chairperson of the set up called Command Post. This was replicated in the outbreak Region of Somali and the outbreak Zone of Dollo. Initially these met on a weekly basis and more frequently if required but later when outbreak went down the meetings took place monthly. The country produced weekly updates initially then monthly and SITREPS which were produced monthly. Quarterly monitoring meetings were held with all Regions in attendance but with a focus on the outbreak area of Somali Region. Presented country progress at all TAG meetings as well as TAG Teleconferences. Regarding SIAs, conducted detailed microplanning in a coordinated manner at National, Regional and Zonal levels, in process monitoring was conducted during SIAs and Independent Monitoring as well as lot quality assurance approach (LQAS) were implemented to evaluate the quality of the campaigns. Regular post SIAs implementation evaluation meetings were held starting from the Zonal level to regional and finally at National level.

In Kenya, the National level Steering Committee Chaired by the Director of Medical Services met every week initially and later monthly. The national technical working group with Social mobilization technical and logistics committees was chaired by the Head of the Integrated Disease Surveillance Unit (IDRSU) Ministry of Health. With respect to Somalia, the main coordination was conducted in Nairobi as it was too dangerous to have the office in Mogadishu. There was a Technical working group that linked by video call with the Regional Committees formed inside Somalia that were responsible for operationalizing the plans. Coordination and planning meetings with the Regional personnel were conducted in Nairobi every 3 to 6 months depending on the need. In South Sudan, on the other hand, the main coordination was under the Health Cluster which was overseeing the management of the evolving crisis. This committee met weekly but to feed into this committee there were 3 Polio related working groups, the Technical, the Logistics and the Social Mob groups. At the State and County levels were locally based coordination committees which coopted all the NGOs that worked in Health at the local levels.

## Communication and Social Mobilization

The nature of the outbreaks and many challenges faced by the polio teams, especially in place where antigovernment teams resisted the SIAs required innovative communication approaches to respond to this outbreak. An outbreak communication plan was developed to address these challenges. The plan focused on 5 main strategies: advocacy, mass media/promotion, community engagement/social mobilization, behavior change/ participatory communication, and capacity building^7^. As a consequence of intensified communication and social mobilization activities, the adherence of the Somali population to polio activities has been secured. A high level of awareness and a low level of OPV refusal were reported during SIAs.

Advocacy with Parliamentarians and engagement with the nomadic population leaders were the main strategies employed in Ethiopia. There was also partnership with Islamic Affairs where between September and December 2014, 1,200 Sheikhs and Imams were sensitized, key messages for polio aligned with the Quran and disseminated and qur’anic schools were visited and sensitized. Furthermore, there was the establishment of social mobilization committees at operational level in Somali Region the outbreak Region with 632 committees fully functional between July 2014 and April 2015. There were also efforts to monitor sources of information which showed that the Mega Phones were the most common source of information. Finally there was the production and dissemination of information, education and communication (IEC) materials, production and dissemination of TV and Radio messages.

In Kenya, a social mobilization committee was in place under the technical working group and responsible for enunciating the social mobilization aspects of the response, producing the necessary messages, disseminating them and guiding the lower levels on social mobilization processes. At the Regional level in the outbreak area this committee was replicated with a very strong component of the locally based NGOs interested in health, in particular the American Red Cross, CORE Group as well as Medicin San Frontier (MSF). This committee operated with its base in Dadaab camps where the outbreak was centered. Kenya was also hit with the vaccine controversy which was precipitated by the Catholic Church. The Communications group addressed this through holding National State Holder Forum, high level Advocacy with the Deputy President, producing newspaper supplements explaining the safety of the vaccines, disseminating messages through media where 26 radio and TV stations aired up to 326 messages and 58 spots in 13 languages all aimed at easing the worry about the safety of the vaccines. They also used a polio survivor who is the Polio Ambassador in Kenya to advocate for continuation of the vaccinations. All this happened at the height of the outbreak and campaigns.

The Somalia social mobilization activities were also coordinated from the Nairobi Office. The main stay of the social mobilization was the networks that were formed locally to implement the social mobilization activities. As at Nov 2014, 3,323 Community Mobilizers, 127 District Social Mobilizers and 21 Regional Social Mobilizers had been identified and trained. As part the work of the network developed, Advocacy was conducted reaching 1,364 Religious leaders, 1,543 Mosques, 1,745,779 persons by phone text messages (SMS), 5, 259 villages reached through sound trucks mounted with mega phones and 3,231 Madarasas were visited and mobilized for vaccination. Nomads were reached through collecting contact details of the nomad elders and this effort saw 2,106 elders details collected and used to truck the nomad movement for vaccination.

South Sudan Social mobilization activities targeting the 32 High Risk counties were organized and coordinated at the National level. The outbreak area was also the most insecure due to conflict. Advocacy with State and County leaders was conducted; announcements were made in Churches and Mosques in local languages (Dinka, Nuer, Arabic and Shiluk). Similarly information, education and communication (IEC) materials were produced in the same local languages. Training materials to support the capacity building at local level were also produced. In Bentiu Camp where the outbreak occurred, 125 Community Mobilizers were identified and trained as a result they sensitized 10,000 households in the camps and surrounding areas. In the rest of the 32 counties, 566 Social Mobilizers were identified and trained. These sensitized over 56,000 households towards embracing vaccinations.

## Discussion

The 2013 outbreak in the HoA was unprecedented. By the time the response was over in 2016, a total of 223 wild polioviruses type1 were reported in 3 countries Somalia (199), Ethiopia (10) and Kenya (14). Additionally, two circulating vaccine derived polioviruses type 2 (cVDPV2) were reported in South Sudan. The polio outbreak in HoA then constituted a significant threat to the WHA-endorsed targets of stopping all forms of poliovirus transmission in the world^9^. The outbreak also signaled the susceptibility of security-compromised populations which is worsened by the weak healthcare systems in the Region^15^. Low population immunity to WPV increases risk of importation and sustained transmission as long as polio eradication is not completed^7^.

Just prior to the outbreak, WPV outbreak risk assessments conducted using existing polio risk assessment methods on a regular basis by the programme^16–18^ placed countries in the HoA at their worst immunity profile within the last 5 years. The risk of WPV importation in Somalia, for instance was compounded by several factors, namely: “(1) for >4 years, an estimated half million children aged <5 years residing in 39 districts of South and Central Somalia have not been reached with large-scale immunization because of a ban on immunization activities imposed by antigovernment elements; (2) provision of routine immunization services is suboptimal as a result of >2 decades of civil unrest; and (3) persistent insecurity in most parts of the country limits the ability of polio officers to access key locations to support, monitor, or evaluate the implementation of SIAs and other polio eradication activities”^7^.

The earlier detection of circulating VDPV (cVDPV) from 2008 to 2013, in these countries was a pointer to a permissive environment, suitable for WPV importation and circulation. Given these precarious realities, the 2013-2016 outbreaks in the HoA were expected and did not come as a surprise. The only surprise is that the teams seemed to have been caught unprepared. The rapid expansion of the outbreak in Somalia both nationally and internationally confirmed this original fear. The outbreak in Somalia expanded not just nationally, but spread to Ethiopia and Kenya in a lightning speed to give what became recognized as the outbreak in HoA.

Three months into the course of the outbreaks, an outbreak response assessment was conducted to evaluate the quality and adequacy of outbreak response activities put in place for the interruption of further transmission within a within 6 months of detection of the first case, in line with the WHA-established standards. The results of the assessment revealed among other things, that the initial response to the outbreak was fast and aggressive; initial investigation and activation of local response were done within 72 hours of index case confirmation. It noted that in Somalia, for instance, the first round of the SIA response was implemented within 5 days of notification of the index case in Banadir, well within the WHA Resolution 59.1 outbreak response requirements in polio-free countries^7^. This initial response in these countries was each followed by intensive rounds of SIAs at 3 to 4-week intervals, targeting various age groups, including adults. The outbreak response was facilitated by committed local governments, local communities and partner organizations. This resulted in marked reduction of the transmission of the polioviruses.

In summary, the 2013-2014 polio outbreaks in the HoA signaled the precarious nature of the achievements of the polio eradication initiative as well as the urgent need to complete polio eradication. Failure to mount an appropriate outbreak response in inaccessible districts posed a major threat to the control of the outbreak, with the risk of continued exportation of WPV from inaccessible to accessible districts in the respective countries and of course internationally. This is thus a lesson to countries facing similar public health challenges or falling into instability and outbreaks of infectious diseases as is the case in the Democratic Republic of Congo. With rapid deterioration of healthcare systems and immunization service delivery, it becomes a high risk for emergence and re-emergence of polioviruses with sustained transmission. In the case of the HoA such experience with polioviruses will no doubt truncate the gains made in polio eradication globally. The HoA has demonstrated its ability to effectively interrupt the transmission of poliovirus, prevent further outbreaks. A consistent adherence to high-quality polio eradication strategies, support of polio eradication partners, persistent research and implementation of innovative strategies to address the large population immunity gap, and a hope of improvement of the political situation in the country will be key determinants of their success^7^.

## Figures and Tables

**Table 1:** Population by age group and country

Country	Total Population	<15 Pop	U5 Pop
DJIBOUTI	978,786	297,173	127,242
ERITREA	6,094,096	2,151,006	609,410
ETHIOPIA	92,399,731	43,011,518	11,087,968
KENYA	46,520,423	20,410,340	8,671,407
SOMALIA	10,638,454	7,033,820	2,330,885
SOUTH SUDAN	12,309,000	5,355,638	3,446,520
SUDAN	39,106,000	16,742,325	7,117,292
UGANDA	39,243,174	20,987,690	7,848,635
TANZANIA	61,415,507	11,054,791	26,408,668
YEMEN	28,063,033	11,438,541	5,331,976
HoA Block	336,768,204	138,482,842	72,980,003

**Table 2:** Number of reported AFP and Wild Poliovirus cases by country, 2013-16

Country	2013	2014	2015	2016	Grand Total
Djibouti	7	3	3	3	16
Eritrea	63	61	68	99	291
Ethiopia	1178(9*)	1211(1*)	1193	1048	4630
Kenya	649(14*)	749(0*)	626	564	2588
Somalia	546(194*)	420(5*)	281	316	1563
South Sudan	294	321(2**)	331	323	1269
Sudan	410	442	436	509	1797
Uganda	493	579	600	717	2389
Tanzania	702	794	799	984	3279
Yemen	614	578	537	715	2444
HoA Block	4956	5158	4874	5278	20266

* Wild polioviruses

** Circulating vaccine derived poliovirus type 2 (cVDPV2)

## Abbreviations: Polio outbreak Investigation and response in Horn of Africa

HoA: Horn of Africa
AFP: Acute Flaccid Paralysis
WPV: Wild Poliovirus
OPV: Oral Polio Vaccine
OPV3: 3^rd^ dose of Oral Polio Vaccine
GPEI: Global Polio Eradication Initiative
WHA: World health Assembly
VDPV: Vaccine Derived Poliovirus
cVDPV2: Circulating Vaccine Derived Poliovirus type 2
KEMRI: Kenya Medical Research Institute
SIAs: Supplementary Immunization Activities
SNIDs: Sub-National Immunization Days
NIDs: National Immunization Days
PTVs: Permanent and Transit Vaccination points
HRT: Hard to Reach areas
CHD: Child Health Days
SIADs: Short Interval Additional Doses
RRM: Rapid Response Mission
TAG: Technical Advisory Group
SITREP: Situation Report
LQAS: Lot Quality Assurance
IDRSU: Integrated Disease Surveillance Unit
NGOs: Non-Governmental Organizations
TV: Television
IEC: Information, Education and Communication
MSF: Medecins Sans Frontiere (Doctors without Borders)
SMS: Phone Text Message
